# *Ficus carica* Polysaccharides Promote the Maturation and Function of Dendritic Cells

**DOI:** 10.3390/ijms150712469

**Published:** 2014-07-14

**Authors:** Jie Tian, Yue Zhang, Xiaomin Yang, Ke Rui, Xinyi Tang, Jie Ma, Jianguo Chen, Huaxi Xu, Liwei Lu, Shengjun Wang

**Affiliations:** 1 Department of Laboratory Medicine, the Affiliated People’s Hospital, Jiangsu University, Zhenjiang 212002, China; E-Mails: tjj850913@163.com (J.T.); einachang@163.com (Y.Z.); j827864988@163.com (K.R.); xinyitang0301@sina.com (X.T.); cjg02@126.com (J.C.); 2 School of Medical Science and Laboratory Medicine, Jiangsu University, Zhenjiang 212013, China; E-Mails: zjflmj19780723@126.com (J.M.); xuhx@ujs.edu.cn (H.X.); 3 School of Food and Biological Engineering, Jiangsu University, Zhenjiang 212013, China; E-Mail: zjyxm2003@hotmail.com; 4 Department of Pathology and Centre of Infection and Immunology, the University of Hong Kong, Hong Kong 999077, China; E-Mail: liweilu@hkucc.hku.hk

**Keywords:** polysaccharide, dendritic cells, dectin-1, Syk, immunomodulators

## Abstract

Various polysaccharides purified from plants are considered to be biological response modifiers and have been shown to enhance immune responses. *Ficus carica* L. is a Chinese traditional plant and has been widely used in Asian countries for its anti-tumor properties. *Ficus carica* polysaccharides (FCPS), one of the most essential and effective components in *Ficus carica* L., have been considered to be a beneficial immunomodulator and may be used in immunotherapy. However, the immunologic mechanism of FCPS is still unclear. Dectin-1 is a non-toll-like pattern recognition receptor, predominately expressed on dendritic cells (DCs). Activation of DCs through dectin-1 signaling can lead to the maturation of DC, thus inducing both innate and adaptive immune responses against tumor development and microbial infection. In our study, we found that FCPS could effectively stimulate DCs, partially through the dectin-1/Syk pathway, and promote their maturation, as shown by the up-regulation of CD40, CD80, CD86, and major histocompatibility complex II (MHCII). FCPS also enhanced the production of cytokines by DCs, including IL-12, IFN-γ, IL-6, and IL-23. Moreover, FCPS-treated DCs showed an enhanced capability to stimulate T cells and promote T cell proliferation. Altogether, these results demonstrate that FCPS are able to activate and maturate DCs, thereby up-regulating the immunostimulatory capacity of DCs, which leads to enhanced T cell responses.

## 1. Introduction

Various plants have been widely used in the treatment of cancer for several decades. Bioactive compounds isolated from *Taxus brevifolia*, *Angelica gigas*, *Catharanthus roseus*, and *Campototheca acuminata* have been applied in the prevention or treatment of different malignancies [1]. Among different active compounds, polysaccharides (PS) are one of the most efficient. PS, which are commonly used in Chinese herbal medicine, have been used for centuries all around the world due to their medicinal benefits, safety, and lack of side-effects. PSs show several beneficial effects, including anti-oxidant [2,3], anti-tumor [4,5], anti-inflammatory [2], anti-diabetic [6,7] and hypolipidemic [8]. Furthermore, they have immune regulatory functions [9], and this strong potential as immunomodulators contributes to their wide clinical application [10].

*Ficus carica* L. is a kind of deciduous tree, which belongs to the *Moraceae* family. Figs, the fruit of *Ficus carica* L., have been used as medicine and food for centuries. The reason for their wide application is the fact that they contain various bioactive compounds, which have been used in the prevention and treatment of several diseases. The best known and most potent fig-derived substances are polysaccharides, denoted *Ficus carica* polysaccharides (FCPS), which have been proven to have anti-tumor and anti-oxidant properties [11,12,13]. However, the mechanism by which they exert their bioremedial effect remains elusive.

Dendritic cells (DCs) are essential initiators of immune responses, which bridge the innate and adaptive immunity. DCs express various receptors critical for the innate immune response, which are known as pattern recognition receptors (PRRs) [14]. Indeed, plant-derived polysaccharides specifically bind to a variety of macrophage cell-surface receptors, including CD14, CR3, Toll-like receptors (TLR), scavenger receptor, and dectin-1 [15]. Although the TLR family crucially contributes to the development of immune responses, the non-TLR PRRs, especially dectin-1, have been considered to play an essential role in mediating polysaccharide-induced activation of DCs, which leads to the maturation of DCs and further induces the activation of the adaptive immune response.

In our study, we demonstrate that FCPS are able to activate and maturate DCs, partially via the dectin-1 pathway, and then promote the secretion of various inflammatory factors by DCs. Furthermore, FCPS are able to increase the immunostimulatory capacity of DCs, thus leading to the activation and proliferation of effector T cells. Therefore, FCPS can be applied as an immunostimulator to enhance immune responses.

## 2. Results

### 2.1. FCPS Activates Syk through the Dectin-1 Pathway in Bone Marrow Dendritic Cells (BMDCs)

**Figure 1:**
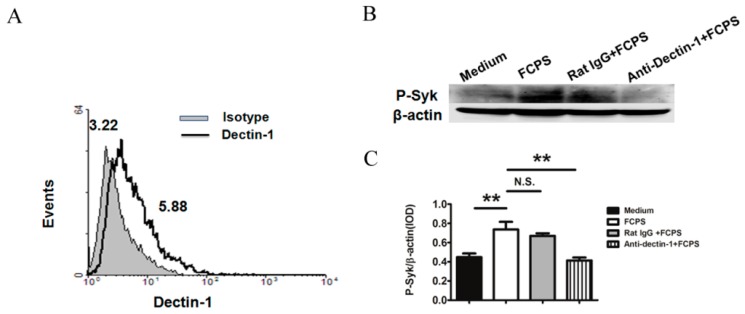
FCPS activate Syk via dectin-1 in BMDCs. (**A**) Expression of dectin-1 on BMDCs. Anti-dectin-1 antibody (thick line) or rat IgG2b (solid gray) were used to stain dectin-1 on BMDCs and then analyzed by flow cytometry; (**B**,**C**) Syk was activated after FCPS stimulation via the dectin-1 pathway; (**B**) Cells were stimulated with FCPS (100 μg/mL) and dectin-1 was blocked with an anti-dectin-1 antibody (5 μg/mL). Cells were lysed and P-Syk levels (upper band) were analyzed by western blot with β-actin as a loading control (lower band); (**C**) Quantitation of the P-Syk/β-actin ratio. Results are shown as means ± SD from threeindependent experiments. ** *p* < 0.01, N.S. no significance.

First, we measured the expression of dectin-1 in BMDCs. Flow cytrometric analysis showed that BMDCs expressed dectin-1 (Figure 1A). The geometric mean fluorescence intensity (GeoMFI) of dectin-1 on BMDCs was 5.52 ± 0.43, while the isotype was 2.97 ± 0.35.

Dectin-1/Syk activation has been reported to play an essential role in certain polysaccharides-induced signal transduction. To investigate whether treatment with FCPS induced dectin-1 expression in BMDCs and whether the downstream signaling molecule Syk was activated as a consequence, phospho-Syk (P-Syk) levels were measured after FCPS stimulation. As shown in Figure 1B,C, the phosphorylation level of Syk dramatically increased upon FCPS treatment. To further confirm whether the activation of Syk was mediated by dectin-1, an anti-dectin-1 antibody was used to block its action. The results showed that P-Syk expression was reduced to a significantly lower level, which indicates that FCPS induces the activation of Syk via dectin-1.

### 2.2. FCPS Promote the Activation and Maturation of BMDCs

**Figure 2:**
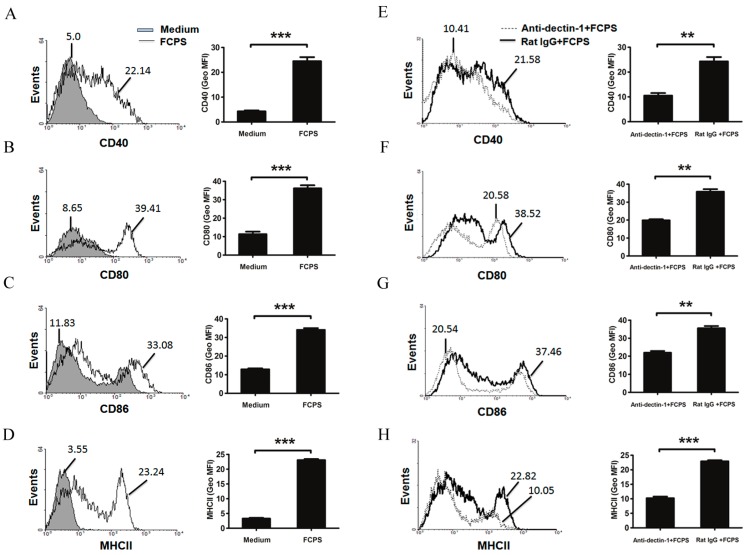
FCPS activate and maturate DC, partially through the dectin-1 pathway. (**A**–**H**) BMDCs were stimulated with FCPS (100 μg/mL) with (**E**–**H**) or without (**A**–**D**) anti-dectin-1 antibody or ratIgG (5 μg/mL) for 48 h. Cells were stained with specific Abs against CD40, CD80, CD86, and MHCII, and then analyzed via flow cytometry. The values shown in the histograms are geometric mean fluorescence intensities (GeoMFI). Results are shown as means ± SD from three independent experiments. *** *p* < 0.001, ** *p* < 0.01.

To evaluate the effect of FCPS treatment on DCs *in vitro*, BMDCs were stimulated with FCPS (100 μg/mL) and the expression of the co-stimulatory molecules CD40, CD80, CD86, and major histocompatibility complex II (MHCII) was analyzed. As indicated in Figure 2A–D , the expression of CD40, CD80, CD86, and MHCII was significantly increased after FCPS stimulation. Similar results were also observed in the D2SC/1 cell line (Supplementary Figure S1). However, after inhibition of dectin-1, up-regulation of the co-stimulatory molecules was partially reversed, while the control IgG was not (Figure 2E–H). This suggests that FCPS could promote the activation and maturation of DCs, partially via the dectin-1 pathway.

### 2.3. FCPS Enhance the Expression of Multiple Cytokines Secreted by DCs

**Figure 3:**
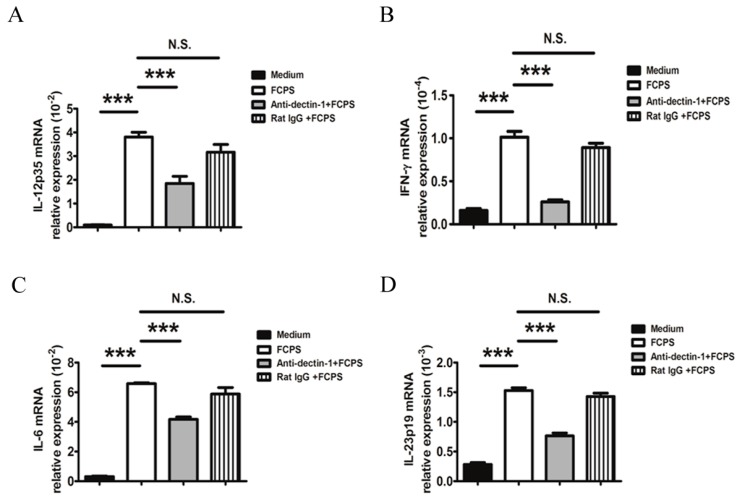
FCPS alter the expression of multiple cytokines secreted by BMDCs. (**A**–**D**) BMDCs were stimulated with FCPS (100 μg/mL) for 24 h with or without anti-dectin-1 antibody or rat IgG (5 μg/mL). Cells were collected and mRNA levels of IL-12p35 (**A**); IFN-γ (**B**); IL-6 (**C**); and IL-23p19 (**D**) were measured by qRT-PCR. All data are shown as means ± SD from three independent experiments. *** *p* < 0.001, N.S. no significance.

Next, we investigated the expression of different cytokines secreted by DCs upon FCPS treatment, including IL-12p35, IFN-γ, IL-6, and IL-23p19. As shown in Figure 3, the mRNA expression levels of IL-12p35, IFN-γ, IL-6, and IL-23p19 were dramatically enhanced. The data suggest that FCPS are able to promote the activation of DC and modify the expression of various cytokines.

**Figure 4:**
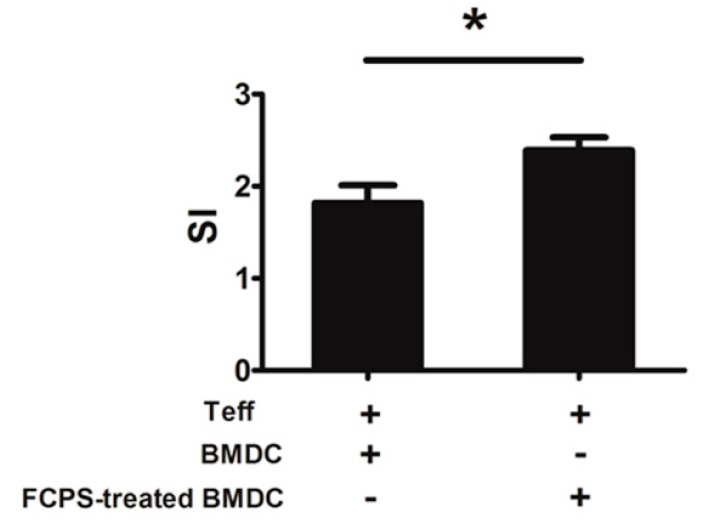
FCPS enhances the immunostimulatory capacity of DCs. BMDCs were stimulated with FCPS (100 μg/mL) for 48 h. Cells were harvested and treated with mitomycin C and then co-cultured with CD4^+^CD25^−^ Teff cells in the presence of anti-CD3 mAb for 72 h. The proliferation was measured via the MTT assay. Results are presented as means ± SD from three independent experiments. * *p* < 0.05.

### 2.4. FCPS Augments the Immunostimulatory Capacity of DCs

Having observed that FCPS could enhance the activation and maturation of BMDCs, we further investigated the capability of DC to stimulate CD4^+^CD25^−^ T cells. CD4^+^CD25^−^ T cells were co-cultured with FCPS-treated BMDCs or non-treated BMDCs in the presence of anti-CD3 mAb. As shown in Figure 4, the proliferation of CD4^+^CD25^−^ T cells was efficiently increased in the presence of FCPS-treated BMDCs, which suggests that FCPS-treated BMDCs obtained a stronger capacity to stimulate and activate effector CD4^+^ T cells.

## 3. Discussion

Cancer is one of the leading causes of death in the world. Although various Western drugs and newly developed tools have been applied in the treatment of cancers, severe adverse effects, especially cell toxicity in non-tumor cells, and growing tolerance by cancers are still major problems in clinical medicine [16]. One of the most promising alternatives in cancer treatment is the use of immunomodulators to enhance host immunity. However, many anti-tumor substances are immunosuppressive agents, which repress tumor growth, but concomitantly adversely affect the immune system. Consequently, investigating novel anti-cancer strategies that improve the host defense response are highly relevant to reduce the therapeutic strain on the patient. It is well known that many traditional oriental medicines have great potential to modulate the immune response and enhance anti-tumor capacity of the organism, such as *Astragalus membranaceus*, *Ganoderma lucidum*, *Angelica sinensis* and so on.

Polysaccharides widely exist in the algae, plants, and microorganism, such as fungi and bacteria. Polysaccharides isolated from natural sources have been shown to be able to regulate the immune system and have great potential as immunomodulators with wide clinical applications [10]. For instance, polysaccharides purified from many mushrooms were shown to promote the activation and maturation of macrophages and dendritic cells, thereby stimulating powerful anti-tumor immune responses [17,18,19,20]. In addition, β-glucans are polysaccharides extracted from the cell wall of fungi, bacteria, or plants. They are well-known biological response modifiers (BRMs), which have been applied to treat cancer for many centuries [21,22,23]. Moreover, polysaccharides from various traditional medicinal herbs have been demonstrated to be immunopotentiating both *in vitro* and *in vivo* [24,25,26]. In our study, FCPS are polysaccharides extracted from *Ficus carica* L., and have been proven to have anti-oxidant and anti-tumor properties, which can be used as an immunomodulator in a clinic setting to augment the immune response. Although many previous studies have demonstrated FCPS’ immunostimulating capacity, the mechanism of this effect is still unclear. Our study is the first to show that FCPS are able to activate DCs, partially via the dectin-1/Syk pathway, and promote their maturation, which leads to an increased production of inflammatory factors, augmenting T cell responses.

Dectin-1 is a natural killer (NK)-cell-receptor-like C-type lectin, which is considered to be involved in the innate immune response to fungal pathogens. It is a non-toll-like pattern recognition receptor and is predominately expressed on myeloid cells, including monocytes/macrophages, dendritic cells, neutrophils, and a subset of T cells. This transmembrane signaling receptor mediates various cellular responses, including antigen binding, uptake, and induction of cytokine production and chemokinesis [27]. Previous studies have shown that engagement of dectin-1 by β-glucan can trigger a series of intracellular signal transduction reactions through the Syk kinase and Raf-1 signaling pathways. This induces a variety of cellular responses, including cell maturation, ligand uptaken by endocytosis and phagocytosis, and the production of cytokines and chemokines, such as IL-6, TNF-α, IL-23, and CXCL2 [27,28,29]. In our study, we found that FCPS stimulated BMDCs partially via dectin-1 signaling to up-regulate the expression of CD40, CD80, CD86, and MHCII, and promote the maturation of DCs. Moreover, as shown in Figure 3, activation of dectin-1 led to a significantly enhancement of the production of multiple cytokines. In addition to the Th1-polarizing cytokines, IL-12 and IFN-γ, FCPS induced the production of the Th17-promoting cytokines in BMDC, IL-6 and IL-23. However, whether FCPS treatment is also able to induce Th1 and Th17 cells *in vivo* remains to be investigated. Priming DCs allows a flexibility in stimulating various cell types, including the regulation of T cell responses. As depicted in Figure 4, FCPS-treated BMDCs significantly augmented the proliferation of effector CD4^+^ T cells, which suggests that the FCPS could enhance the immunostimulating capacity of DCs.

## 4. Experimental Section

### 4.1. Mice

Pathogen-free male C57BL/6 mice were obtained from Yangzhou University (Yangzhou, China). Mice were used at 6–8 weeks of age. All experiments were approved by the Institutional Committee on the Use of Animals for Research and Teaching.

### 4.2. FCPS

FCPS were isolated and purified from the pomace of *Ficus carica* L., as previously described in more detail [13]. FCPS is ashen powder, soluble in water and insoluble in methanol, ethanol, acetone, ether, and other organic solvents. FCPS mainly consist of pyranopolysaccharides. The molecular weight distribution was 5.92 × 10^5^−1.95 × 10^6^. FCPS contain >91% polysaccharides, and no nucleic acids and proteins. The lipopolysaccharide contamination was removed and the endotoxin level was 0.06 EU/mL, as tested by the gel-clot method (Associates of Cape Cod, Inc., East Falmouth, MA, USA).

### 4.3. Cell Line

The D2SC/1 cell line is a retrovirally immortalized dendritic cell line from BALB/c mouse spleen that was generously provided by Will van Ewijk, Netherlands. Cells were cultured in DMEM (GIBCO, Carlsbad, CA, USA), supplemented with 10% heat-inactivated newborn calf serum (GIBCO, Carlsbad, CA, USA), 2 mM l-glutamine, penicillin/streptomycin and 50 mM β-mercaptoethanol at 37 °C in a humidified atmosphere with 5% CO_2_.

### 4.4. Bone Marrow-Derived DC (BMDC)

BMDCs were generated following a previously described method [30]. Briefly, bone marrow cells in femurs and tibiae were flushed out with complete RPMI 1640 medium using a 1 mL syringe. The complete RMPI 1640 medium was prepared by adding 10% heat-inactivated newborn calf serum (NCS) (GIBCO, Carlsbad, CA, USA), 50 µM 2-mercaptoethanol and penicillin/streptomycin to RPMI 1640 medium (GIBCO). BM cells were seeded into 6-well flat bottom plates (Costar, Corning, NY, USA) at a density of 2.5 × 10^6^/mL in complete RPMI 1640 medium, supplemented with granulocyte-macrophage colony-stimulating factor (GM-CSF, 10 ng/mL) (Clongene Biotech, Hangzhou, China) and IL-4 (PeproTech, Rocky Hill, NJ, USA; 10 ng/mL). Cells were cultured at 37 °C. 5% CO_2_ for 48 h and the non-adherent cells were washed out. Subsequently, fresh medium supplemented with GM-CSF was added. On day 5, half of the medium was replaced with fresh GM-CSF-supplemented medium. On day 7, the non-adherent cells were harvested for further experiments.

### 4.5. Western Blot Analysis

Western blot analysis was performed as described previously [31]. Briefly, proteins were extracted and then separated by sodium-dodecyl-sulfate-polyacrylamide gel electrophoresis (SDS-PAGE), transferred onto immobilon PVDF membranes (Bio-Rad, Hercules, CA, USA), and challenged with rabbit phospho-Syk antibody (CST, Danvers, MA, USA) and mouse β-actin antibody (Abcam, Cambridge, UK). Signals were visualized with chemiluminescence detection reagents (Champion Chemical, Whittier, CA, USA).

### 4.6. RNA Isolation and Quantitative Real-Time PCR (qRT-PCR)

BMDCs were stimulated in 24-well flat-bottom plates. In some conditions, BMDCs were pretreated with anti-dectin-1 mAb or rat IgG (5 μg/mL) for 1 h at 37 °C and then treated with 100 μg/mL FCPS. After 24 h stimulation, total RNA was isolated and reversed-transcribed with ReverTra Ace qPCR RT kit (TOYOBO, Osaka, Japan). IL-12p35, IFN-γ, IL-6, and IL-23p19 mRNA levels were quantified by qRT-PCR amplification using Rotor-Gene 6000 (Corbett Life Science, Sydney, Australia). Briefly, cDNA was amplified in a 25 μL reaction mixture containing 12.5 μL SYBR Premix Ex Taq (1×) (Takara, Dalian, China), 200 nM of each primer, 100 ng of cDNA using the recommended cycling conditions. The sequences for the primers used are: IL-12p35 sense primer, 5'-TGACATGGTGAAGACGGC-3', IL-12p35 antisense primer GCCTGGAACTCTGTCTGGTA-3'; IFNγ sense primer, CGCTACACACTGCATCTTGG, IFNγ antisense primer, 5'-TGAGCTCATTGAATGCTTGG-3'; IL-6 sense primer, 5'-GGCCTTCCCTACTTCACAAG, IL-6 antisense primer, 5'-ATTTCCACGATTTCCCAGAG-3'; IL-23p19 sense primer, 5'-TGACATGGTGAAGACGGC-3', IL-23p19 anti-sense primer, 5'-GCCTGGAACTCTGTCTGGTA-3'; β-actin sense primer, 5'-TGGAATCCTGTGGCATCCATGAAAC-3', β-actin antisense primer, 5'-TAAAACGCAGCTCAGTAACAGTCCG-3'. Each gene was amplified in triplicate and cDNA concentration differences were normalized to β-actin.

### 4.7. Flow Cytometry

Single cell suspensions were prepared and then stained with relevant fluorochrome-conjugated anti-CD40, anti-CD80, anti-CD86, anti-MHCII mAbs (eBioscience, San Diego, CA, USA). Anti-dectin-1 (Invivogen, San Diego, CA, USA) and FITC-conjugated goat anti-rat IgG (KPL, Gaithersburg, MD, USA) were used to detect dectin-1 expression. HB197 supernatant was used for blocking Fc receptors. Cells were stained with the aforementioned antibodies in PBS for 30 min at 4 °C. Cells were analyzed using a FACSCalibur flow cytometer (Becton Dickinson, Sparks, MD, USA) and data were analyzed using WinMDI 2.8 software (Joseph Trotter, http://facs.scripps.edu/software.html).

### 4.8. In Vitro Proliferation Assays

CD4^+^CD25^−^ T cells were isolated from wild-type C57BL/6 mice splenocytes with a CD4^+^ T cell negative selection kit (Invitrogen, Carlsbad, CA, USA), FITC-conjugated anti-CD25 (BD Pharmingen, San Diego, CA, USA) antibody and anti-FITC microbeads (Miltenyi Biotec, Bergisch Gladbach, Germany). The purity of CD4^+^CD25^−^ T cells was >90% (data not shown). BMDCs were pretreated with FCPS or not for 48 h, and then cells were harvested and treated with mitomycin C (Kyowa, Tokyo, Japan; 50 μg/mL) for 20 min. Subsequently, 5 × 10^4^ CD4^+^CD25^−^ T cells were co-cultured in triplicate in flat-bottom 96 wells with 1 × 10^4^ mitomycin C-treated BMDCs in the presence of 10 μg/mL anti-CD3 mAb. Cells were incubated for 72 h and the proliferation was measured by MTT assay kit (Beyotime, Shanghai, China). Data from the co-culture were expressed as stimulation index (SI) = OD in stimulated culture/OD in unstimulated culture.

### 4.9. Statistics

Results were expressed as means ± SD. The statistical significance of differences between groups was determined by the Student’s *t* test or two-way analysis of variance. All analyses were performed using SPSS11.5 software (SPSS, Chicago, IL, USA). Differences were considered significant at a *p* level less than 0.05.

## 5. Conclusions

In conclusion, our data demonstrate that FCPS could effectively activate DCs, partially via dectin-1/Syk signaling, promote the maturation of DCs, and induce the production of multiple cytokines. Moreover, FCPS significantly enhanced the immunostimulating capacity of DCs and therefore augments T cell responses. Thus, FCPS may be useful as an immune stimulant and a promising candidate as a potential medicine with low toxicity. Taken the effective and safe profiles together, our data also provide support for potential future clinical studies on applying this ancient Chinese traditional medicine.

## Supplementary Files

Supplementary File 1
