# Supplementary material for: Ficus carica Polysaccharides Promote the Maturation and Function of Dendritic Cells
Source: Int J Mol Sci. 2014 Jul 14;15(7):12469–79. doi: 10.3390/ijms150712469 (PMC4139854; doi:10.3390/ijms150712469)

Supplementary Information

Figure S1. FCPS promote the maturation of D2SC/1 cells. (**A**–**D**) D2SC/1 cells were stimulated with FCPS (100 μg/mL) for 24 h. Cells were stained with specific Abs against CD40 (**A**); CD80 (**B**); CD86 (**C**); and MHCII (**D**), and then analyzed via flow cytometry. The values shown in the histograms are geometric mean fluorescence intensities (GeoMFI). The data were representative of three independent experiments.

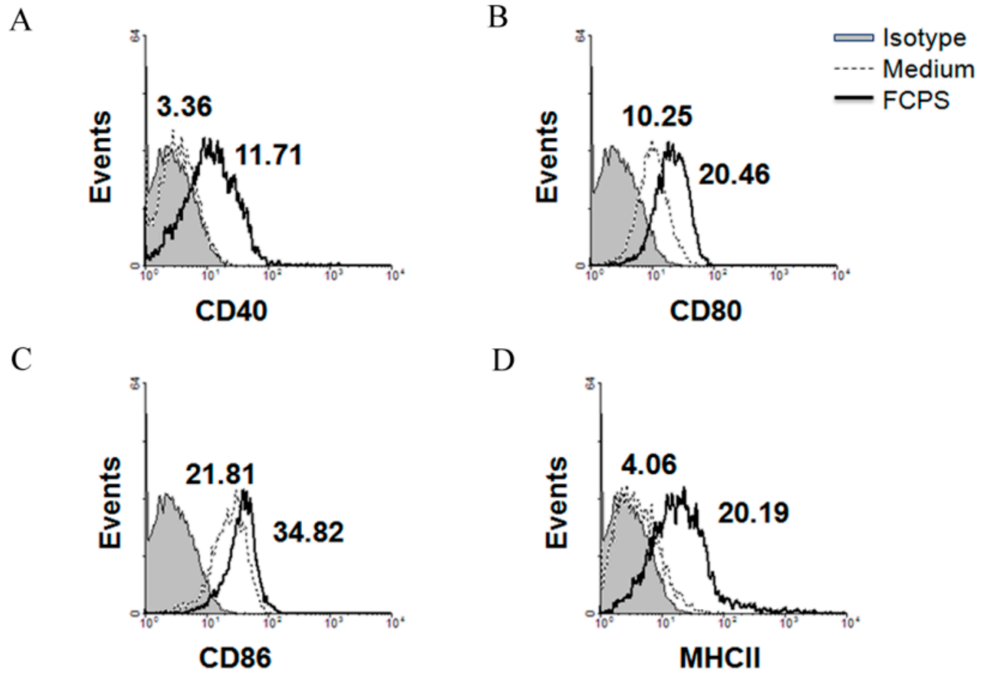

Supplement: Supplementary File 1 [file ijms-15-12469-s001.pdf]
